# Research, diagnosis and education in inborn errors of metabolism in Colombia: 20 years’ experience from a reference center

**DOI:** 10.1186/s13023-018-0879-2

**Published:** 2018-08-16

**Authors:** Olga Y. Echeverri, Johana M. Guevara, Ángela J. Espejo-Mojica, Andrea Ardila, Ninna Pulido, Magda Reyes, Alexander Rodriguez-Lopez, Carlos J. Alméciga-Díaz, Luis A. Barrera

**Affiliations:** 1 Institute for the Study of Inborn Errors of Metabolism, Faculty of Science, Pontificia Universidad Javeriana, Cra. 7 No 43 - 82, Building 54, Room 305A, Bogotá, Colombia; 2 Clinical Laboratory – Inborn Errors of Metabolism Section, Hospital Universitario San Ignacio, Bogotá, Colombia; 3 Clínica de Errores Innatos del Metabolismo, Hospital Universitario San Ignacio, Bogotá, Colombia

**Keywords:** Inborn errors of metabolism, Colombia, Latin America, Research, Education, Diagnosis, Training, Rare diseases

## Abstract

The use of specialized centers has been the main alternative for an appropriate diagnosis, management and follow up of patients affected by inborn errors of metabolism (IEM). These centers facilitate the training of different professionals, as well as the research at basic, translational and clinical levels. Nevertheless, few reports have described the experience of these centers and their local and/or global impact in the study of IEM. In this paper, we describe the experience of a Colombian reference center for the research, diagnosis, training and education on IEM. During the last 20 years, important advances have been achieved in the clinical knowledge of these disorders, as well as in the local availability of several diagnosis tests. Organic acidurias have been the most frequently detected diseases, followed by aminoacidopathies and peroxisomal disorders. Research efforts have been focused in the production of recombinant proteins in microorganisms towards the development of new enzyme replacement therapies, the design of gene therapy vectors and the use of bioinformatics tools for the understanding of IEM. In addition, this center has participated in the education and training of a large number professionals at different levels, which has contributed to increase the knowledge and divulgation of these disorders along the country. Noteworthy, in close collaboration with patient advocacy groups, we have participated in the discussion and construction of initiatives for the inclusion of diagnosis tests and treatments in the health system.

## Background

Inborn errors of metabolism (IEM) is a group of monogenic diseases with low frequency that constitute a challenge for health professionals due to their clinical, genetic and biochemical heterogeneity. In fact, to assure an appropriate diagnosis and management, it is ideal to have specialized centers that bring together technical and professional resources. The main work models for this kind of centers are those developed by the European Union where many countries articulate to optimize resources in benefit of the patient [1–3]. These centers facilitate patient’s care and follow up, as well as knowledge sharing among different professionals, which is particularly useful in the cases of very rare diseases and difficult clinical cases. In addition, this system facilitates clinical and basic research and allows training programs for professionals within and outside the network [1–3].

In Latin America, diagnosis and research in IEM, and other rare diseases, is widely affected by variant and complicated economic, politic, geographic and social context of this region. In fact, economic and technical resources are not equally distributed among countries. Furthermore, most of Latin American countries are considered developing countries, which implies that economical resources must be invested to overcome social and health challenges such as malnutrition, access and quality of public services, basic education, unemployment and international external debts, among others [4]. In addition, difficulties in geographical access to some areas and border policies limit communication and collaboration among specialized centers in some cases. Moreover, the tropical location of most countries establishes infectious diseases as a health priority [4]. All these circumstances greatly vary among countries within the region requiring individual national efforts that have proven successful in different areas, such as the establishment of full coverage newborn screening (NBS) for small molecule diseases in countries like Costa Rica, Cuba and Uruguay; and the development of clinical and research specialized centers in Brazil, Argentina and Mexico [5–7]. In 1996 it was created the Latin American Society for Inborn Errors and Neonatal Screening (SLEIMPN) allowing the integration of professionals working in NBS and IEM along Latin American countries, the interchange and cooperation between members, and the training and education of health and non-health professionals. In addition, this organization has promoted the development of reference centers and the necessity to implement quality control standards for diagnosis and NBS tests.

Colombia is a developing country of around 50 million inhabitants with a complex geography, located near the equator in the north west of South America. Health system coverage reaches more than 97% of the population, but quality and sustainability are challenges to be faced. In general, IEM and rare diseases were practically absent from health programs and policies until about 10 years ago. An ambitious law in favor of orphan diseases was issued in 2010 but its implementation is advancing slowly. The current situation is that Colombia is one of the countries in Latin America with the biggest number of patients being treated with enzyme replacement therapy and with a very rapid increase in diagnosis and treatment of organic acidemias, aminoacidopathies and neurological diseases. In terms of NBS only hypothyroidism is actively searched as part of a national funded program, while the IEM are mainly diagnosed after clinical onset. On the other hand, the research in IEM is led by academic institutions [6, 8, 9]. However, while the current trends and debates on diagnosis, treatment and research for IEM in developed countries are widely published (e.g. NBS, gene therapy, international collaboration, among others), limited information is found about these topics in developing countries. In this article, we describe 20 years of experience in diagnoses, research, training, education and social advocacy in IEM in Colombia from a reference center (Instituto de Errores Innatos del Metabolismo -IEIM-, Pontificia Universidad Javeriana, Bogotá D.C.). It is important to note that there are other centers in Colombia working in diagnosis and research of IEM such as Centro de Investigaciones en Bioquímica (Universidad de los Andes, Bogotá D.C.), Instituto de Genética Humana (Pontificia Universidad Javeriana, Bogotá D.C.), Instituto de Genética (Universidad Nacional de Colombia, Bogotá D.C.), Centro de Investigaciones en Anomalías Congénitas y Enfermedades Raras (Universidad Icesi, Cali), and Grupo de Medicina Genómica y Metabolismo (Fundación Cardiovascular de Colombia, Floridablanca, Santander).

We consider that the information presented in this review will contribute to the knowledge of a broad spectrum of the situation of IEM in the context of a country without NBS and where metabolic testing is mainly performed by private institutions. In addition, we consider that this kind of reports will encourage other laboratories to share their experiences, which might facilitate the identification of common strategies and challenges from closer scenarios and contribute to the development of public policies and the consolidation of new reference centers.

## Biochemical diagnosis

### Biochemical testing for diagnosis of IEM

**Fig. 1:**
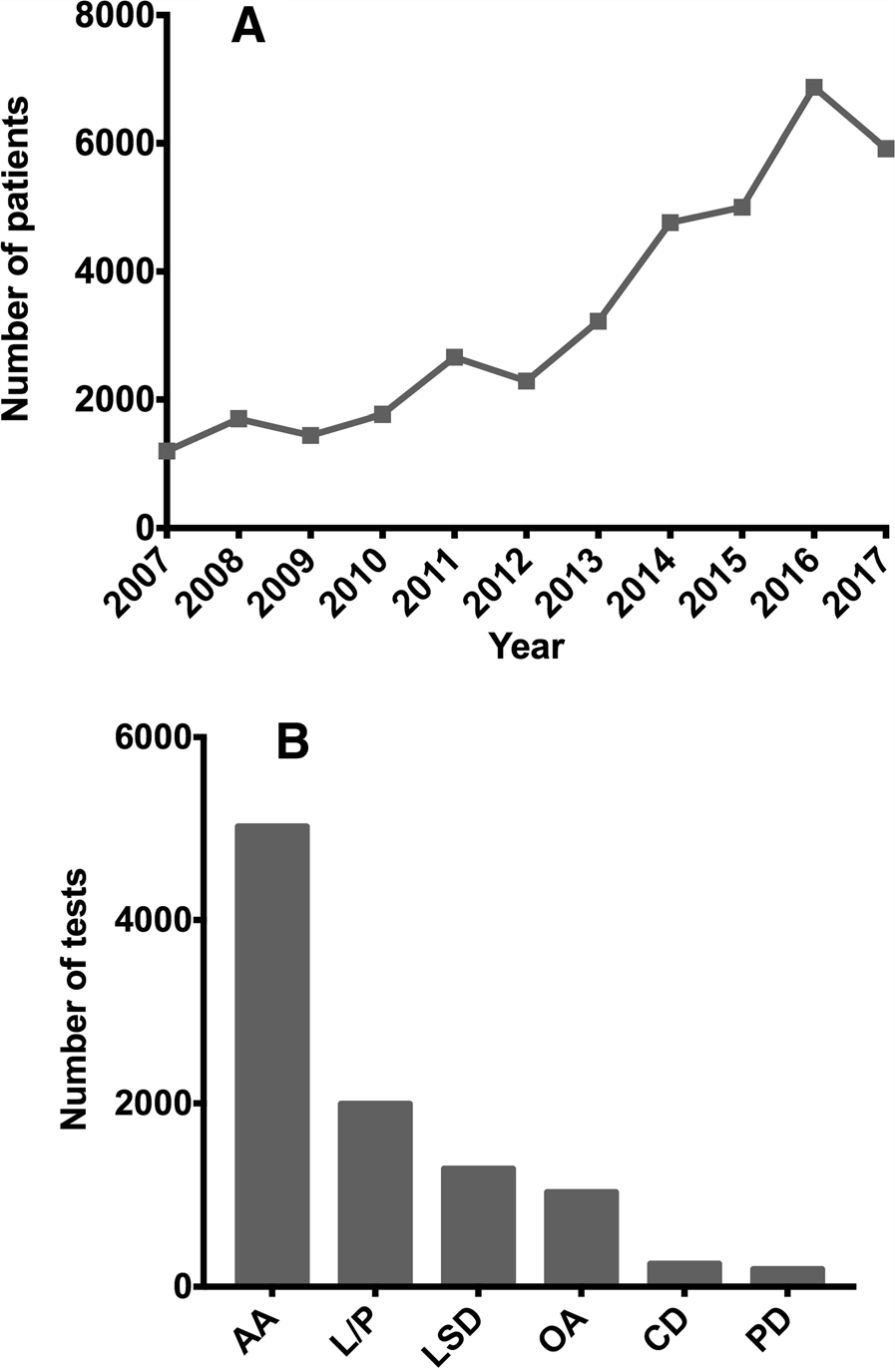
Diagnostic tests processed in the reference center. **a** Number of patient samples analyzed in the last 10 years. **b** Distribution of requested tests according to clinical suspicion in the last 10 years. AA: aminoacidopathies; OA: organic acidurias; LSD: lysosomal storage disorders; CD: carbohydrate disorders; PD: peroxisomal diseases (Total tests = 9772)

Since the establishment of the IEIM a primary goal has been to provide biochemical tools to improve the diagnosis of IEM in Colombia. This center was among the first institutions in the country offering diagnostic tests for IEM. Twenty years ago, the diagnostic service began offering qualitative tests for diagnosis of aminoacidopathies and defects of monosaccharides metabolism, as well as enzymatic assays for some lysosomal storage diseases (LSD). Nowadays, the diagnostic service offers tests that include the biochemical confirmation of aminoacidopathies (amino acids quantitation by high performance liquid chromatography, HPLC), organic acidurias (gas chromatography–mass spectrometry, GC-MS), most common LSD and neurodegenerative diseases, among others [10–18]. Summed to the increase in the tests offered, it has been observed that the number of tests processed have increased from 1203 samples in 2007 to 5915 in 2017 (Fig. 1a). This behavior has been mainly influenced by the improvement in the clinician’s knowledge about these disorders and the local availability of confirmatory tests. It is also important to consider the availability of specific treatment for several disorders, which in most of the cases can change the natural course of the disease (e.g. phenylketonuria, propionic aciduria, isovaleric aciduria, among others).

The above-mentioned circumstances constitute an important progress in the diagnosis of IEM in Colombia, since currently the diagnosis is carried out under clinical suspicion in high-risk population. In fact, in Colombia NBS is performed only for congenital hypothyroidism, while for other metabolic disorders it is available on demand and generally performed outside the country, limiting the accessibility to such biochemical tools. Recently, some private institutions are performing screening tests for diagnose amino acids and organic acidurias through tandem mass spectrometry. Nevertheless, these diagnostics analyzes are carried out selectively, which limits the generation of relevant epidemiological data [19, 20]. In the case of small molecule disorders (i.e. aminoacidophaties, organic acidurias and galactosemia, among others), this situation contrasts with that observed in developed countries where NBS has been stablished since 60’s [5]. In Latin America, different schemes of NBS are available depending on the country. For instance, Costa Rica has universal expanded NBS with high population coverage [5, 6, 21–23], while Uruguay, Cuba, Brazil, Chile and Mexico have selective NBS for common treatable IEM (i.e. phenylketonuria, biotinidase deficiency, galactosemia, maple syrup urine disease -MSUD-, and congenital adrenal hyperplasia, among others) and some of them have regional or private access to expanded NBS [6, 21, 24–29]. Screening of IEM in other Latin American countries is not universal and it is mainly provided by private institutions [6, 21, 30–33].

Currently, our center performs the analyses for aminoacidopathies, organic acidurias (OA), carbohydrates disorders, lysosomal storage disorders, and peroxisomal diseases (Fig. 1b). Additionally, we performed other supporting tests like lactic-pyruvic ratio and glucose-6-phosphate dehydrogenase, which together correspond to 21% of the total processed samples. In the period of 2007 to 2017, 36,858 samples were processed, being those related with aminoacidopathies diagnosis the most frequently requested tests (Fig. 1b). It is difficult to compare this data with other experiences, due to limited available reports from countries with circumstances similar to those observed in Colombia. For instance, evidence from middle east countries and Brazil cannot be directly compared, since those countries perform diagnosis panels (including different biochemical tests for a group of diseases) to any patient fulfilling certain clinical criteria suggestive of an IEM [34–39]. In contrast, other experience, like Lebanese, Indian, Cuban and Brazilian ones, report findings of targeted screening in high-risk population [40–42]. To the best of our knowledge, the closest experience published so far was made by an Egyptian group that reported a similar pattern than that observed in our center [43].

**Fig. 2:**
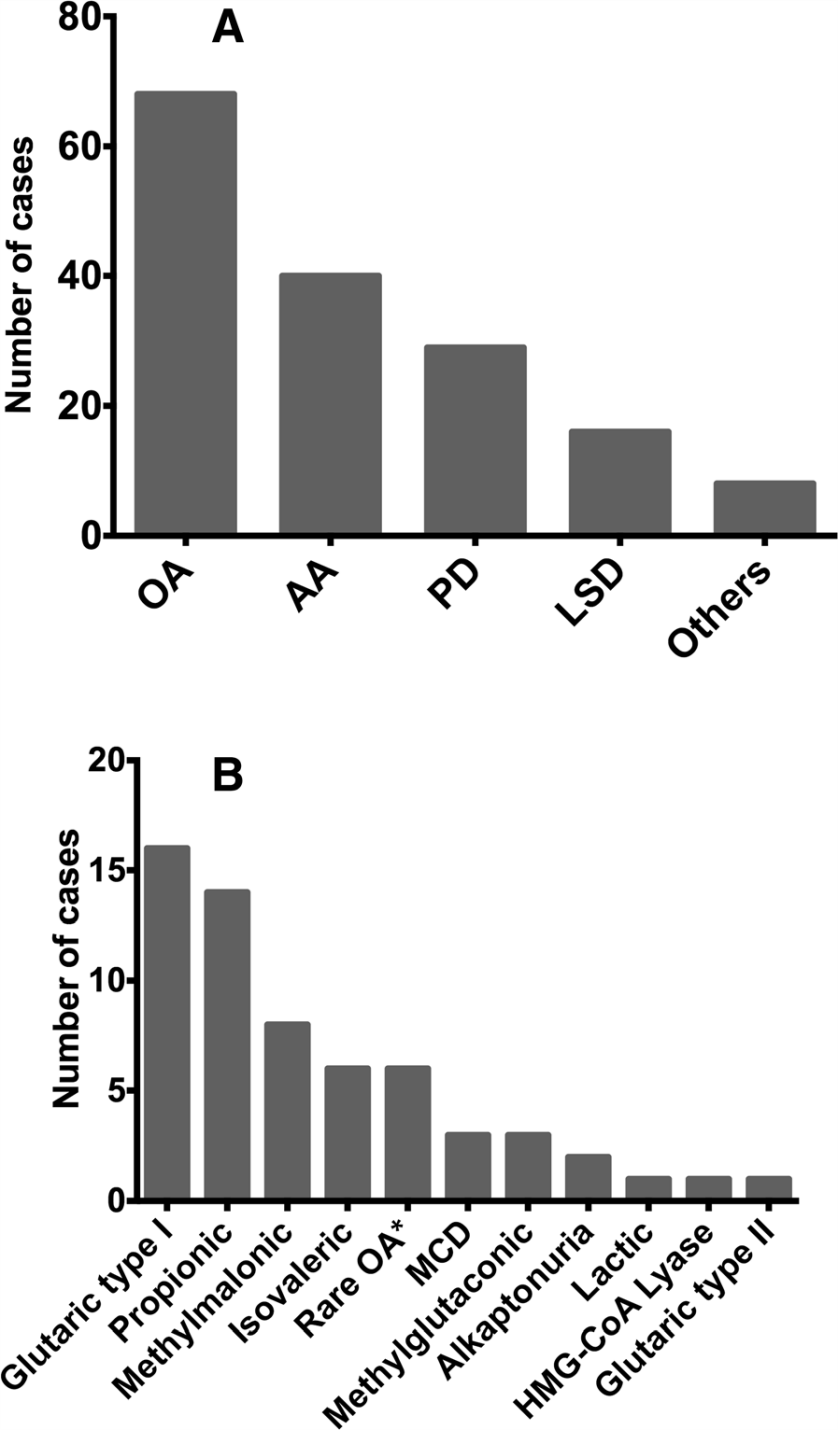
IEM detected in the reference center. **a** Diagnosis made according to biochemical-cellular classification of IEM. Data include diagnosis made in the period 2007–2017. **b** Organic acidurias diagnosed during the last 10 years. These data correspond to cases detected with typical biochemical profiles detected by GC-MS. AA: Aminoacidopathies; LSD: Lysosomal storage disorders; MCD: Multiple carboxylase deficiency; OA: Organic acidurias; PD: Peroxisomal diseases

Among the diagnosed cases, OA are the most frequently detected diseases with 81 cases confirmed, corresponding to 49% of total performed diagnosis, followed by aminoacidopathies (20%) (Fig. 2a). These results contrast with the experiences reported by Brazilian groups, where aminoacidopathies represent around 20% of the diagnosed cases and OA account for less than 10%. Moreover, for those laboratories, LSD are the most frequent diagnosis representing more than 45% of total cases [44, 45]. Comparisons with other reports from Latin America is difficult considering that those reports are focused only on the diagnosis of aminoacidopathies and OA. For instance, Cornejo et al. [29] reported 63% of confirmed OA versus 37% of aminoacidopathies. However, it is important to note that PKU was not included in this report since it is detected by NBS. Besides, Ibarra-González et al. [26] found in Mexican population a similar number of cases diagnosed with OA and AA, while Queiruga in 2015 reported that AA was the most frequent group detected in Uruguay, followed by congenital hypothyroidism, congenital adrenal hyperplasia and cystic fibrosis [24]. In addition, although most of the worldwide available literature is based on NBS scenarios, it is also observed a high heterogeneity in the detected IEM depending on the studied region [24, 46–50]. This data remarks the high variability in the IEM detection capability of each country, which might be influenced by circumstances such as: 1) particular interests and experience of each center, 2) technology available for screening and confirmatory tests, 3) accessibility to testing by national or private programs, 4) timeframe of each report, and 5) genetic variations among populations.

The most frequent OA observed in our center are glutaric aciduria type I (26%) and propionic aciduria (23%) (Fig. 2b). On the other hand, methylmalonic acidurias, which have been reported as very frequent in Asian populations [40, 51], correspond only to 13% of the detected OA. In contrast, OA reported as very rare corresponded to about 8% of the identified cases (Fig. 2b), including 2-hydroxyglutaric aciduria, piroglutamic aciduria, and succinyl-CoA dehydrogenase deficiency [52]. As observed in Table 1, this behavior is similar to that reported for Chile; while reports from Brazil and Cuba show high differences in the frequency of glutaric, propionic, methylmalonic and isovaleric acidemias. Moreover, the high frequency observed for glutaric aciduria type I does not coincide with reports available from European and Asian countries [29]. These differences can be associated to the possibility that in the Colombia it is more feasible to detect chronic or progressive diseases, such as glutaric aciduria, than neonatal lethal conditions like the severe forms of propionic and isovaleric acidemias [53].

**Table 1:** Organic acidemias detected in high risk population in Latin America

	Brazil [40]	Cuba [42]	Chile [29]	Colombia
Time frame	15 years	5.5 years	7.5 years	9 years
OA diagnosed	218	46	40	61
Glutaric aciduria type I	33 (14,1%)	0	8 (20%)	16 (26,2%)
Propionic aciduria	18 (7,7%)	3 (6,5%)	11(27,5%)	14 (22,9%)
Methylmalonic aciduria	34 (14,5%)	5 (10,8%)	7 (17,5%)	8 (13,1%)
Isovaleric acidemia	7 (3%)	0	5 (12,5%)	6 (9,8%)

During the period of 2007 to 2017, 40 patients with aminoacidopathies have been diagnosed, being non-ketotic hyperglycinemia (NKHG) the most frequently aminoacidopathy (Table 2). Data from aminoacidopathies detection in high risk population is scarce due to their inclusion in most of the NBS programs. Nevertheless, similar to the results observed for OA, our data differ from those reported for other countries, where PKU, MSUD and urea cycle disorders (UCD) are the most frequent entities [39, 48–50]. Such behavior may be explained by the mixed genetic background of Colombian population. In addition, it is important to highlight that since the diagnosis is done through a symptom-based approach, it is possible to miss those patients with severe phenotypes that lead to early patient death, as it is the case for severe phenotypes of MSUD and UCD [54, 55]. In addition, NBS studies usually do not actively look for some aminoacidopathies like NKHG [56–58].

**Table 2:** Aminoacidopathies detected in the IEIM between 2007 and 2016

	No. Cases	Percentage
NKHG	13	32
MSUD	10	24
UCD	8	20
HPA	7	17
Tyrosinemia	2	5
Homocistinuria	1	2

In addition, our center offers some biochemical tools for other groups of IEM. In this context, in the last 10 years we have diagnosed 16 LSD, mainly mucopolysaccharidoses (MPS) and sphingolipidosis (gangliosidosis GM1 and GM2). In addition, by very long chain fatty acid (VLCFA) analyses we have been able to detect 29 patients with peroxisomal disorders, most of them (93%) corresponding to X-linked adrenoleukodystrophy (X-ALD).

Currently, the diagnostic area is in continuous development, with several challenges that has to be faced. On one hand, as a referral laboratory receiving samples from all over the country, it is difficult to keep track of the clinical histories of the patients and have personal contact with clinicians. Such contact is essential for a adequate following up of the patients, as well as for enhancement of the diagnostic process in complex cases. On the other hand, we are in a constant improvement of the technical and professional resources. Therefore, we look forward to improve the technical tools available for other group of disorders, such as LSD and mitochondrial disorders.

### Interdisciplinary work

Complementary to the work performed in the biochemical diagnosis area, an important work has been done in the construction and consolidation of interinstitutional groups for the discussion and analysis of clinical cases of difficult diagnosis. Within these groups, it has been observed the valuable participation of biochemists, since they offer an important support for the interpretation and clinical correlation of the biochemical diagnostic tests. These types of alliances were established initially with one institution, which progress to at least once meeting per month in three different clinical centers. Finally, as a result of the continuous search to improve the diagnosis of IEM patients, the first inborn errors of metabolism clinic in Colombia was recently funded. This clinic was the first multidisciplinary approach developed in Colombia for the diagnosis and integral management of patients with IEM. During the last 3 years, this initiative has consolidated an important group of physicians from different medical specialties (i.e. child neurologist, geneticist, pediatrician, and child endocrinologist), nutritionists, psychologist and biochemists. This initiative is recognized as one of the reference groups for diagnosis and integral follow up for patients with diagnosis or suspicion of IEM. In addition, the model of attention is being used by other centers to organize similar groups along the country. Currently, the efforts are focused on increasing the time for patient attention and to improve the administrative and clinical resources offered for long term following up of patients, emergency care for critical patients, adult care and nationwide coverage.

In summary, during the last 20 years, we have worked in the consolidation of an integral center for the study of IEM. Specially, this institution has become in a reference center for the diagnosis of IEM of small molecule. Simultaneously, we have given to the medical community the possibility to improve the diagnosis, which allows an early and proper treatment, a follow-up of the patient, and the consolidation of an interdisciplinary work. These results contribute to establish the incidence of this type of entities and to extend the knowledge of the IEM in our population.

## Education and training

One of the main purposes of our center has been to contribute to the clinical training and academic formation in basic, applied and clinical aspects of IEM at all education levels. Academic activities performed for undergraduate students are focused on topics related to biochemistry, IEM and biotechnology through courses mainly addressed to students of basic sciences and health related careers (Table 3). In addition, we offer the students the opportunity to get involved in research activities through internships and development of undergraduate research projects.

**Table 3:** Training programs

Program	Type	Frequency	Students Profile	Intensity (weeks)	Mean of students per year
Training in biochemical diagnosis of IEM	Internship	Permanent	Geneticists (2007)	6/Full time^a^	2
			Pediatric Neurologists (2005)	8/Full time^a^	3
			Pediatricians	4/Part time^b^	Occasional
			Neonatologists	4/Part time^b^	3
Metabolic disease fundamentals	Theoretical Course	Permanent	Undergraduate students basic sciences	4 h per week (17 weeks)	30
Health biotechnology	Theoretical Course	Permanent	Undergraduate students basic sciences	4 h per week (17 weeks)	100
Trainig in biochemical and biotechnological tools	Internship	Permanent	Undergraduate students basic sciences	17 weeks – Minimum 6 h per week depending on student schedule	4
Undergraduate research project	Internship	Permanent	Undergraduate students basic sciences	17 – Part time^b^	2
MSc and PhD	Academic program	Permanent	Graduate students with clinical or basic science background.	2–4 years/Full time^a^	1
Young research program	Internship	On demand	Professionals from basic sciences.	1 year /full time	1
Introduction to IEM for pediatricians.	Theoretical Course	On demand	Pediatricians	8 h/w 3 w	25^c^
Diploma in IEM of small molecule	Theoretical Course	On demand	Health professionals	130 h	23^c^

^a^Between 30 and 40 h per week

^b^Around 20 h per week

^c^Media of student among the courses offered

At graduate level, academic activities on the clinical area involves the training in biochemical tools for diagnostic of IEM for healthcare professionals. Such training also involves different aspects of the biochemistry and physiopathology of these diseases. This kind of training is provided primarily to clinicians of different medical specialties including child neurologists (42%), geneticists (35%), neonatologists (21%) and pediatricians (2%). On the other hand, graduate training is offered to professionals in basic sciences that get involved in basic and applied research in different research lines that include development of new therapies, molecular biology and basic biology of IEM. In addition, we have published two handbooks about clinical aspects of inborn errors of metabolism [59, 60], as well several chapters in pediatrics textbooks [61–64].

## Social advocacy in favor of the inborn errors of metabolism

Colombia is a developing country of 50 million inhabitants, with a health system comprised of a subsidized regime and a contributory regime. In this system, the contributory regime, through the mandatory payroll contributions along with the money from taxes, help to pay the health expenses of the subsidized regime. People belonging to either one system have had access to a specified package of benefits known as POS (obligatory plan of health) [65]. Although the Organization for Economic Co-operation and Development (OECD) recognized that Colombia has made great improvement’s in the health system [66]; rare diseases, and particularly IEM, were not part of the health programs neither private nor public, until very recently. These recent improvements have led to the progressive inclusion of some diagnostic test and treatments as part of the POS.

We led and participated in the organization of a The Colombian Association for Rare Diseases (ACER). The main objectives of ACER, among others, were to represent and work in favor of all rare diseases, but specially the more neglected ones. Later, ACER was included as part of a patient advocacy group (Colombian Federation of Rare Diseases – FECOER), who is currently working for the recognition of these diseases and their patients and families.

In cooperation with patient advocacy groups, we played an important role in the discussions that gave rise to the Orphan Diseases Act (Law 1392 of 2010), for which the orphan diseases are recognized of public interest and norms are adopted to guarantee the social inclusion of the patients and caregivers. This law covers important aspects for orphan diseases, such as: 1) the obligation of the government to establish a national registry of patients with rare diseases, 2) to create a system to import and distribute orphan drugs aiming to get fair access to all the patients, and 3) the establishment of specialized networks for diagnosis, treatment and orphan drugs distribution (specialized pharmacies). This Act also covers the education of human talent on these diseases in all the educational levels. This Act asks the government to stimulate research in prevention and treatment including psychological and psychiatric disorders associated with these diseases, social inclusion, and integration of patients into the society. The law is an advanced piece of legislation, unfortunately, most of the aspects have not been implemented and it has been through the *tutela* mechanism that Colombia has been able to advance in the diagnosis and treatment of those diseases. The *tutela* is a mechanism that protects the fundamental rights and speeds up the legal decisions; and that also protects persons who feels that his or her fundamental rights have been infringed.

The import of orphan drugs has been a problem still not fully solved despite of many advances in the last years. Most of orphan drugs must be imported and the process usually takes up to four to 6 months. The last few years, due to the growing number of diagnosed patients, several companies have started to commercialize these drugs in Colombia and there are now orphan products readily available in the country. In addition, we also participate in the discussions that gave rise to the Decree 481 of 2004, which regulates the processes, requirements and incentives for research, development, production, import and marketing of vital drugs not available in our country. Since many of these vital drugs were also orphan drugs, this norm represented an important contribution for the proper and timely treatment of inborn errors of metabolism.

## Research

Enzyme replacement therapy (ERT) and gene therapy are part of the main alternatives for the treatment of LSD [67]. Our group have worked in the design, development, production and evaluation of proteins and vectors for both type therapies. For ERT we have reported the production and characterization of human recombinant lysosomal iduronate-2-sulfate sulfatase (IDS), N-acetylgalactosamine-6-sulfate sulfatase (GALNS), and β-N-acetylhexosaminidases (Hex-A, Hex-B, and Hex-S) in the bacteria *Escherichia coli* and the yeast *Pichia pastoris* [68–74], as well as the phenylalanine hydroxylase in *Lactobacillus plantarum* [75] (Table 4). In the two first expression platforms, we have evaluated different strains, vectors, and culture conditions [73, 76–80]. All recombinant proteins have shown activity levels similar or higher that those reported for native or recombinant proteins produced in other expression systems, even IDS and GALNS produced in *E. coli* [73, 81]. Likewise, they have shown similar pH and temperature stability profiles compared to proteins produced in mammalian cells or native proteins. In addition, proteins obtained from *P. pastoris* are taken up by cultured cells and delivered to the lysosome in a dose dependent manner through an endocytic pathway, possibly mediated by mannose or mannose-6-phosphate receptors [68, 69, 74], showing the potential of this host to produce therapeutic enzymes for LSD. Recombinant proteins produced in *E. coli* were not uptake by cell lines, which demonstrated that the absence of N-glycosylations are necessary to mediate the cellular uptake of the enzymes but not to produce active or stable lysosomal enzymes [73]. Finally, the use of a genetically modified lactic acid bacteria, as an in situ (i.e. gut) expression system to produce a recombinant phenylalanine hydroxylase (PAH) for the treatment of PKU, showed promising results in the evaluation of a new strategy to facilitate the oral administration of recombinant enzymes for the treatment of IEM [75]. This approach could be used to avoid the intravenous administration of the purified enzyme in ERT [82], improving the patient quality of life, adherence to therapy, and reducing production costs [82].

**Table 4:** Summary of recombinant lysosomal enzymes produced in microorganisms

Enzyme	Disease	Expression system	Culture condition	Enzyme activity (nmol h^− 1^ mg^− 1^)
Iduronate-2-sulfate sulfatase IDS (E.C. 3.1.6.13)	Hunter (MPS II)	*Escherichia coli* K12 JM 109	100 mL	1.2 to 2.8
		*Escherichia coli* DH5α	100 mL	25.9 to 34.2
		*Pichia pastoris* GS115	100 mL	4.2
			1.65 L	29.5
			1.65 L (optimized gene)	49.7
N-acetylgalactosamine 6 sulfate sulfatase GALNS (E.C.3.1.6.4)	Morquio IVA (MPS IVA)	*Escherichia coli BL21* (DE3)	3 L	0.067 to 0.078
			3 L (improved culture conditions)	6.8
			Purified enzyme	2.9
		*Pichia pastoris* GS115	1.65 L	0.02 to 0.09
			1.65 L (coexpression of SUMF1)	16.69
β-N-acetylhexosaminidases Hex-A Hex-B, and Hex-S (E.C.3.2.1.52)	GM2 Gangliosidoses (Tay Sachs and Sandhoff diseases)	*Pichia pastoris* GS115	1.65 L (* AOX * promoter)	Hex-A: 13,124 Hex-B: 12,779 Hex-S: 14,606
			Purified enzymes	Hex-A: 1.35 × 10^6^ Hex-B: 1.27 × 10^6^ Hex-S: 1.39 × 10^6^
			1.65 L (*GAP* promoter)	Hex-A: 32,666
Phenylalanine hydroxylase PAH (EC 1.14.16.1)	Phenylketonuria (PKU)	*Lactobacillus plantarum* CM_PUJ411	100 mL	Active

In terms gene therapy, we have focused on the design and evaluation of vectors for Morquio A disease. Overall, the use of AAV-derived vectors, eukaryotic promoters (elongation factor 1α or α1-antitrypsin) and co-expression of *GALNS* and *SUMF1* genes resulted in a significant increase in enzyme activity and secretion in patient fibroblasts or Morquio A mouse chondrocytes [83–86]. In vivo assays showed that a single intravenous administration allowed GALNS activity up to 20% of wild-type levels plasma and between 3 and 36% of wild-type levels in tissues (Table 5) [87, 88]. Additionally, the AAV vector was modified by insertion of a short acidic amino acid peptide within the viral capsid, to confer affinity of the virus for hydroxyapatite (HA), the major constituent of bone matrix [89]. This engineered vector allowed higher vector genome copies in bone, which led to enzyme activity levels in bone of 42% of those observed in wild-type animals [89].

**Table 5:** Summary of gene therapy in-vivo results

Tissue	AAV-GALNS	AAV-GALNS AAV-SUMF1	Bone-tagged AAV-GALNS
Plasma	8.5	19.4	NA
Liver	21.9	36.6	24.3
Spleen	4.5	5.4	3.2
Kidney	3.2	3.1	1.8
Lung	4.2	4.1	3.0
Heart	6.5	30.6	61.8
Brain	4.0	9.1	35.8
Bone marrow	2.0	10.4	13.8
Bone (leg)	0.2	33.3	41.9

Results are expressed as percentage of wild-type levels. *NA* not assayed

**Fig. 3:**
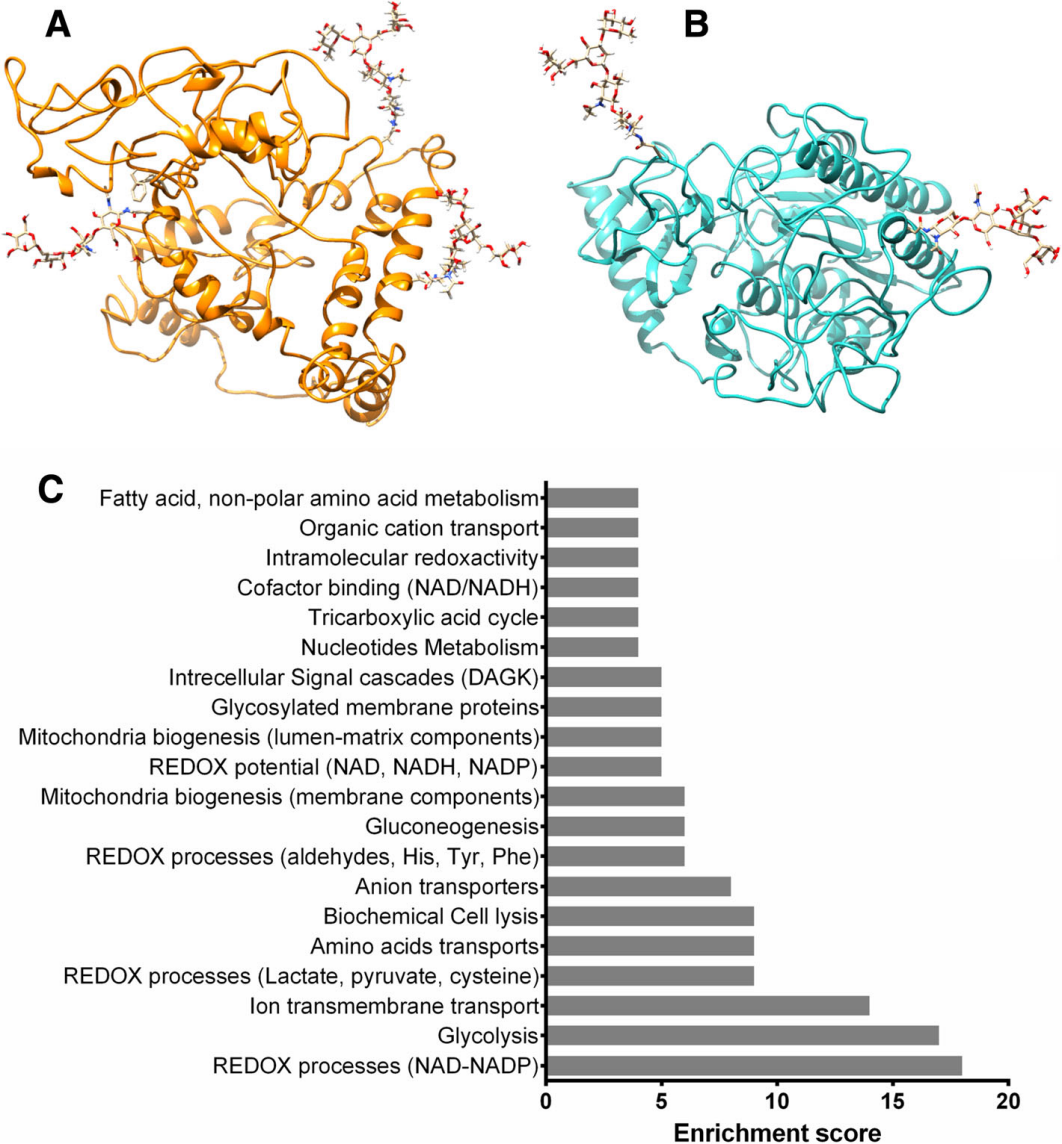
Use of bioinformatics tools for the study of IEM. Tertiary structure of human IDS (**a**) and GALNS (**b**) enzymes were modeled by protein threading based on the reported structure of other lysosomal enzymes. IDS and GALNS 3D models have been used in phenotype-genotype correlation studies as well as for the design of epitope-specific antibodies. N-glycosylations were modeled by using GlyProt at GLYCOSCIENCES.de server. **c** Gene enrichment analysis for impaired cellular process in MPS models, identified by a computational systems biology approach

Bioinformatics studies have involved the study of phenotype-genotype correlations, evolution, modelling of metabolic alterations using an *in-silico* systems biology approach, and mechanical and mechanobiological mathematical models of growth plate. IDS and GALNS were modeled (Fig. 3) allowing the understanding, correlation and prediction of phenotype-genotype correlations, as well as docking and molecular dynamic modeling against natural and artificial substrates [90–92]. Structural modelling of IDS allowed the identification and design of peptides to produce chicken immunoglobulin Y (IgY) anti-IDS antibodies, which were used for the development of an ELISA test [93].

To understand the global metabolic consequences of protein mutation, an *in-silico* systems biology approach was used to identify metabolic pathways impaired in each MPS [94]. The results predicted several commonly affected pathways, including oxidative stress, activation of β-oxidation, synthesis of ROS by NADH dehydrogenase, and cytochrome C oxidase, among others (Fig. 3c) [85]. A similar strategy was used to model the biochemical consequences of Arylsulfatase A (ARSA) deficiency, showing that that mitochondrial metabolism and amino acid transport c the main reactions affected in a glia cell model [95]. These findings allowed us to hypothesize that ARSA deficiency might lead to metabolic consequences that not only compromise the myelin band or the glycosphingolipids metabolism but also the overall metabolic function of the nervous system.

Finally, mechanical and mechanobiological mathematical models were formulated to develop theoretical approximations to understand the growth plate physiology and the pathological changes observed in MPS [96, 97]. The results predicted that the main factors involved in growth plate pathology are the altered cell differentiation and the changes in structure organization [98].

## Conclusions and future remarks

We have described the different contributions from a reference center to the advance in the diagnosis, research, education, training and divulgation of the IEM in Colombia. Noteworthy, the continuous growth of this reference center has had a significant impact in the inclusion and recognition of this group of disorders within the Colombia’s health system. The strategy to achieve this goal has involved the continuous work in the knowledge and divulgation of the biochemical and clinical characteristics of these disorders and their treatment alternatives. The education and training of health and non-health professionals has also played an important role on the recognition of this group of disorders. One important aspect has been the collaborative work and communication between the clinical laboratory and the clinicians, which has been essential for the timely diagnosis of patients, as well as for the enhancement of the diagnostic of complex cases and the follow up of patients. Although significant improvements have been done during the last years in the diagnosis, treatment and follow up of these disorders in Colombia, future efforts should be focus in the decentralization and consolidation of specialized centers, as well as in the construction of knowledge networks, since until now the work in the field has focused on consolidation of individualized centers. In addition, we need to work in the empowerment of different patient advocacy groups, working together to achieve important goals such as the extended newborn screening program [32, 99], the transition to new omics technologies [100], and the establishment of research programs sponsored by the government.

## Abbreviations

AA: Aminoacidopathies
AAV: Adeno-associated virus
ACER: Colombian Association for Rare Diseases
ARSA: Arylsulfatase A
CMV: Cytomegalovirus
ERT: Enzyme replacement therapy
FECOER: Colombian Federation of Rare Diseases
FGE: Formylglycine-generating enzyme
GALC: Galactocerebrosidase
GALNS: N-acetylgalactosamine-6-sulfate sulfatase
GBA: Glucocerebrosidase
GC-MS: Gas chromatography–mass spectrometry
HA: Hydroxyapatite
Hex: β-N-acetylhexosaminidases
HPLC: High performance liquid chromatography
IDS: Iduronate-2-sulfate sulfatase
IEM: Inborn errors of metabolism
IgY: Immunoglobulin Y
LAB: Lactic acid bacteria
LSD: Lysosomal storage diseases
MPS: Mucopolysaccharidoses
MS/MS: Tandem mass spectrometry
MSUD: Maple syrup urine disease
NBS: Newborn screening
NKHG: Non-ketotic hyperglycinemia
OA: Organic acidurias
OECD: Organization for Economic Co-operation and Development
PAH: Phenylalanine hydroxylase
PKU: Phenylketonuria
POS: Obligatory plan of health
SLEIMPN: Latin American Society for Inborn Errors and Neonatal Screening
SNPs: Single nucleotide polymorphisms
SUMF1: Sulfatase modifying factor 1
UCD: Urea cycle disorders
VLCFA: Very long chain fatty acid
X-ALD: X-linked adrenoleukodystrophy
